# Sex differences in morphology across an expanding range edge in the flightless ground beetle, *Carabus hortensis*

**DOI:** 10.1002/ece3.7593

**Published:** 2021-07-01

**Authors:** Elisabeth Yarwood, Claudia Drees, Jeremy E. Niven, Marisa Gawel, Wiebke Schuett

**Affiliations:** ^1^ School of Life Sciences University of Sussex Falmer East Sussex UK; ^2^ Institute of Zoology Universität Hamburg Hamburg Germany

**Keywords:** Body size, expansion front, range expansion, sexual dimorphism, spatial sorting, trait‐dependent dispersal

## Abstract

1. Species’ ranges are dynamic, changing through range shifts, contractions, and expansions. Individuals at the edge of a species’ shifting range often possess morphological traits that increase movement capacity, that are not observed in individuals farther back within the species’ range. Although morphological traits that increase in proportion toward the range edge may differ between the sexes, such sex differences are rarely studied.
2. Here, we test the hypotheses that body size and condition increase with proximity to an expanding range edge in the flightless ground beetle, *Carabus hortensis*, and that these trait changes differ between the sexes.
3. Male, but not female, body size increased with proximity to the range edge. Body size was positively correlated with male front and mid tibia length and to female hind tibia length, indicating that body size is indicative of movement capacity in both sexes. Body condition (relative to body size) decreased with increasing population density in males but not females. Population density was lowest at the range edge.
4. Our results indicate that sex is an important factor influencing patterns in trait distribution across species’ ranges, and future studies should investigate changes in morphological traits across expanding range margins separately for males and females. We discuss the implications for sex differences in resource allocation and reproductive rates for trait differentiation across species’ shifting ranges.

## Graphical abstract

The distribution of morphological traits may change across species' shifting or expanding ranges. Here, we show that the differentiation of such traits is sex‐specific in the ground beetle species *Carabus hortensis*: Male, but not female, body size increased with proximity to the range edge.

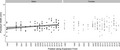

## 1 INTRODUCTION

Species’ ranges are dynamic, flexible, and capable of change through range shifts, contractions, and expansions (Andrewartha & Birch, 1954; Sexton et al., 2009). Range shifts can occur as the result of stochastic processes, whereby random individuals at the edge of the species’ range (hereafter: range edge) slowly expand the range over time through random movements (Skellam, 1951). Yet, range shifts may often be driven by a subset of individuals residing at the range edge, who are characterized by traits that increase capacity for forward movement, not possessed by individuals farther back within the species’ range (Chuang & Peterson, 2016; Phillips et al., 2006; Shine et al., 2011). Such traits are often associated with morphology.

Understanding how traits associated with morphology differ across expanding or shifting ranges is important because such patterns in trait distribution may alter the pace of range changes (Bowler & Benton, 2005; Roff, 1984; Zera & Denno, 1997) and influence population dynamics (*e.g*., Gadgil & Bossert, 1970; Stearns, 1976). Intraspecific (Bolnick et al., 2003) and interspecific (Rudolf, 2007) interactions, including predator–prey interactions (Cohen et al., 1993), resource use (Polis, 1984), and individual capacity to overcome environmental change (Huey & Kingsolver, 1989), may also be affected by trait differentiation at range edges. In invasive species, traits associated with morphology that propel the species forward could amplify the negative effects that the invader has upon native flora and fauna (Phillips et al., 2006). Hence, traits that drive range shifts at range edges can have large‐scale ecological impacts.

Differences in traits associated with morphology among individuals from the center or “core” of a species’ range versus the range edge have been documented across different taxa (*e.g*., Berthouly‐Salazar et al., 2012; Bonte et al., 2012; Brandner et al., 2013; Hill et al., 1999; Phillips et al., 2006). For example, across homogenous environments, individual body condition may increase toward the range edge (*e.g*., Brown et al., 2013) where population densities and associated competition for resources are typically low. Individuals that have increased locomotor capacity (*i.e*., those with longer legs (Phillips et al., 2006), increased flight muscle mass, and/or wing size (Heidinger et al., 2018; Hill et al., 1999)) also increase in frequency with proximity to the range edge. Patterns in trait distribution across species’ ranges may occur through (a) trait‐dependent dispersal (*e.g*., Heidinger et al., 2018), whereby only individuals with traits that confer the highest dispersal capacity disperse to the range edge (Heidinger et al., 2018); (b) phenotypic plasticity to environmental variation (*e.g*., Tejedo Madueño et al., 2010), in which individuals plastically respond to environmental differences in the core versus the edge of the range; or (c) the process of spatial sorting (Phillips et al., 2008; Shine et al., 2011), whereby genes that improve movement propensity become sorted in space, such that individuals with a greater capacity for forward movement reach the range edge at a time where the only available mates are similarly adapted individuals. Assortative mating (Fisher, 1919) then occurs at the range edge.

We may observe a stronger gradient in the distribution of traits associated with movement in one sex over the other, if species’ range shifts are primarily driven by one sex (Berthouly‐Salazar et al., 2012). Such sex biases in movement may arise because (a) sexual traits selected for in males and females are often divergent due to fundamental differences in male and female reproductive investment (Bateman, 1948; Darwin, 1871; Maynard Smith, 1978) where males generally maximize reproductive fitness through increasing mating opportunities, while female reproductive success depends on egg and offspring production (Trivers, 1972); and (b) some sexually dimorphic traits, such as behavior or body size, affect movement capacity and may therefore enhance the propensity for one sex to disperse (Bowler & Benton, 2009). Strong sexual disparities in trait distribution across species’ ranges may be especially prominent in scramble‐competition type mating systems, in which males evolve adaptations that improve locomotion to increase mate searching efficiency (Husak & Fox, 2008). Although sex differences in morphological traits at range edges have been observed in a few studies, results differ and studies are largely restricted to vertebrates (*e.g*., Bodden & Puschendorf, 2019; Campbell & Echternacht, 2003; Gunnarsson et al., 2012; Miller et al., 2017; Simberloff et al., 2000, but see Laparie et al., 2013 for a study on an insect species). This may be explained by differences in reproductive and mating systems across the different taxa. Consequently, further investigation of patterns in trait distribution across species’ ranges in taxa with different reproductive and mating systems is required if we are to fully understand the mechanisms underlying morphological differentiation across species’ shifting ranges.

Here, we study sex‐specific changes in body size and body condition along an expanding range margin of the flightless ground beetle, *Carabus hortensis* L., at its western distribution edge in northern Germany. Previous monitoring of this population (Völler et al., 2018) allows us to pinpoint the precise range edge of the species in previous years, meaning that traits of individuals from the center or “core” of the range can be systematically compared to traits of individuals from the range edge. We predict that individuals at the range edge should be larger in body size than those from the “core”, if body size is directly related to leg length and associated movement capacity. We test this prediction, assessing the correlation between leg length and body size.

Male *C. hortensis*, like other *Carabus* species (e.g., Drees & Huk, 2000; Weber & Heimbach, 2001), are generally more active than females (Szyszko et al., 2004). Because males of other *Carabus* species are known to actively search for females with whom to mate (Turin et al., 2003), male *C. hortensis* activity may be an adaptation to increasing mate searching capacity. Male *C. hortensis* are therefore likely to be the more dispersive sex owing to mate searching behavior (Turin et al., 2003). Thus, we predict that the change in body size across the *C. hortensis* range will be stronger in males than females, with body size increasing toward the range edge.

We further predict that population density will decline with proximity to the expansion front. Thus, we predict that, as long as conditions at the range edge are suitable, and population density is lower at the range edge than at the core, individuals will have better body condition at the range edge than at the core of the species’ range, owing to reduced intraspecific competition for resources (Brown, 1984).

## 2 MATERIALS AND METHODS

### 2.1 Study species, trapping, and maintenance

*Carabus hortensis* Linnaeus, 1758 (Coleoptera, Carabidae) ground beetles were studied from August to September 2018 in the Lüneburger Heide, Lower Saxony, Germany, where the species has expanded its range westward from ancient forests into adjacent forested areas at a constant pace over the last 25 years (Völler et al., 2018).

To sample individuals from the range edge and from regions farther back in the species’ range (*i.e*., across the expansion front), rows of live pitfall traps (hereafter “trap rows”) were installed parallel to the most westerly edge of the species’ range, starting from the leading edge of the expansion and spanning across 3 km to the point at which the species was first observed in this area in 1995 (Völler et al., 2018). A map of the study site with some trap rows included in our study can be found in Völler et al. (2018). Habitat across the sample area consisted of coherent forests of coniferous, broad‐leaved, and mixed stands, and there were no clear systematic habitat differences across the expansion front. We installed 17 trap rows, which reflected the positions of the *C. hortensis’* westerly range edge for the years 1995, 1999, 2001, 2003, 2005, and 2007–2018. Because *C. hortensis* has dispersed westward by approximately 130 m each year (Völler et al., 2018), an additional trap row was placed 130 m beyond “trap row 2018” where we expected beetles to arrive the following year, in 2019: “trap row 2019”. This trap row mainly served to assess whether *C. hortensis* had expanded its range further than expected and to ensure that, if it had, we would catch those individuals. Thus, we installed 18 trap rows in total. Each trap row contained 12 live pitfall traps that were separated by 10 m to span 120 m. To ensure that beetles were caught at the range edge where population densities were expected to be low, 12 additional pitfall traps were positioned at each of the five most westerly trap rows, such that trap rows 2015–2019 contained 24 traps and all other rows contained 12 traps. We found beetles at “trap row 2018”, which was the expected range edge when our study took place in 2018, but not “trap row 2019.” This suggested that *C. hortensis* were still expanding their range westward by at least 130 m per year, but not as far as 260 m per year.

Live pitfall traps (10 cm diameter, 500‐ml plastic cup inside) were dug into the ground so that they were level with the surface soil. A drainage tube around the cup served as a structural support and water drained through holes in the bottom of each trap. A metal mesh cover prevented small vertebrates, leaves, and sticks from falling into the traps. All traps were baited with a piece of cellulose soaked in red wine and were emptied and rebaited once every 7–8 days (Schuett et al., 2018).

The total number of individuals caught at each trap row from August to September 2018 was used as a proxy for population density (Baars, 1979). However, because sampling efforts at each trap row differed depending on whether the trap row contained 12 or 24 pitfall traps, the population density at each trap row was divided by the total number of pitfall traps present in that row and this was divided by the total number of days over which each trap row was sampled. This provided the number of beetles caught per trap and trapping day for each trap row, which was used as a proxy for population density. The female‐to‐male sex ratio at each trap row was also quantified, by dividing the total number of females caught at each trap row by the total number of males caught at that trap row. When more than 30 individuals (15% of the cases) were caught at a particular trap row in one week, we did not record their sex. Consequently, our measure of sex ratio is only an estimate in these cases.

Individuals were either taken to the laboratory for further studies or released to the site of capture. Released individuals were marked using permanent marker pens (Edding 781, Edding International GmbH, Ahrensburg, Germany), to avoid retesting upon capture. Each week, where possible (based on the number of individuals in a trap row), the body size and mass were measured for four females and four males selected randomly from each trap row. In total, 161 female and 92 male *C. hortensis* were weighed to the nearest milligram (CA‐103 Phoenix Instrument, Phoenix Instrument GmBH, Garbsen, Germany). Individual pronotum width was then measured as a proxy for body size. Pronotum width has previously been used as a proxy for movement ability in other studies of flightless carabid beetles (*e.g*., Laparie et al., 2013), because it describes the space available for locomotor muscles (Berwaerts et al., 2002). Dorsal photographs of each individual were taken over a laminated page of mm grid paper using a camera phone (Wileyfox Swift 2X, Wileyfox), and the widest section of the pronotum was later measured to the nearest 0.1 mm, using ImageJ (Schneider et al., 2012). To assess our prediction that pronotum width was indicative of movement capacity, we later measured the leg lengths of retained specimens. The tibia and femur of the front, mid, and hind leg from the left‐hand side of each beetle were carefully removed and mounted upon a piece of card using insect glue. Photographs of each leg were taken using a digital camera (Canon EOS 7D; Canon, Tokyo, Japan) mounted on a stereoscopic microscope (Nikon SMZ‐U; Nikon Corp., Tokyo, Japan), and the length of each front, mid, and hind tibia and femur was measured to the nearest 0.1 mm using ImageJ. Two photographs taken of each leg showed that the measurements were significantly repeatable. Each leg length was then calculated as the mean of the two measurements.

Individual body condition scores (relative mass to body size, in g) were calculated separately for males and females. Several different methods to obtain measures for body condition exist, including taking direct measurements of energy stores (*e.g*., Weatherhead & Brown, 1996), calculating body condition from the residuals from reduced major axis regressions of body mass versus body size (Green, 2001), and calculating body condition as the residuals from ordinary least‐squares (OLS) regression of body mass versus body size (*e.g*., Cordero et al., 1999; Dobson et al., 1999). Here, we employ the latter, more commonly used method, using residual scores from a linear model (LM) of body mass against pronotum width (males: y = 0.027 * x + 0.295 g, *R* ^2^ = 0.096, *F*_1,90_ = 9.673, *p* =.003, *N* = 92; females: y = 0.030 * x + 0.377 g, *R* ^2^ = 0.087, *F*_1,159_ = 15.360, *p* <.001, *N* = 161) to calculate body condition. We note, however, that, as with other methods, calculating body condition by this means is not without its caveats. For example, several assumptions must be made to permit calculation of body condition from OLS mass/body size residuals (outlined in: Green, 2001). Moreover, some variation in body condition calculated via OLS mass/body size residuals may be attributed to intraspecific variation in lean dry body mass (*e.g*., Schulte‐Hostedde et al., 2005), meaning that OLS mass/body size residuals may somewhat inaccurately describe lipid stores and therefore body condition (Moya‐Laraño et al., 2008).

### 2.2 Statistical analysis

All statistical analyses were carried out using R version 3.3.2 (R Core Team, 2019). We performed Spearman's rank correlations (Spearman, 1904) to assess the relationships between body condition, body size, and body mass, and between body size and leg lengths. Spearman's rank correlations were used because not all data followed a normal distribution. Body condition and body size were not highly correlated (*R*_s_ < 0.3; Table S1). Body condition and body mass were highly positively correlated (*R*_s_ > 0.9), and body mass and body size were significantly positively correlated in both male and female *C. hortensis* (Table S1). To avoid multicollinearity, only data concerning individual body size and body condition were analyzed further. Our measures of pronotum width were significantly positively correlated with leg length (Table S1). To corroborate our hypothesis that male *C. hortensis* may be the more dispersive sex, owing to mate searching behavior (Turin et al., 2003), we performed additional Spearman's rank correlations to assess the relationship between the female‐to‐male sex ratio at each trap row and position along the expansion front.

We predicted that *C. hortensis* population density should decline with increasing proximity to the range edge. The effect of position along the expansion front upon population density was determined using a LM, with population density as the response variable and the position along the expansion front as the explanatory variable. Position along the expansion front was a discrete variable, in which the positions of the *C. hortensis* westerly range edge for the years 1995, 1999, 2001, 2003, 2005, and 2007–2018 were the values (“trap row 2019” was excluded from analyses as no beetles were caught there), 2018 was the range edge, and the greatest distance was between 2018 and 1995 (Völler et al., 2018). For the purpose of analysis, the position along the expansion front was treated as a continuous variable.

To determine whether individuals from different positions along the expansion front differed in their body size and body condition, we performed linear mixed models (LMMs) with body size and body condition as the response variables and the position along the expansion front as the main explanatory variable. The week (week 1 to week 6) in which individuals were collected was included as a random term. In 34% of the cases, beetles were collected from traps from which at least one other beetle was collected in the same week. To account for any potential interdependence of beetles collected from the same trap on the same week, the trap from which individuals were collected nested within the week of collection (week 1 to week 6) was included as a second random term. Again, the position along the expansion front was treated as a continuous variable during analyses. Population density was included in the models as a covariate (Tables 1 and 2). To test whether the relationships between body size/body condition and position along the expansion front differed between the sexes, we added the explanatory variable of sex as well as its interaction with position along the expansion front.

**TABLE 1:** Summary of test statistics from LMMs with the pronotum width as a proxy for body size as a response in males and females (M + F), females alone (F), and males alone (M)

Response Variable	Sex	Random Term	Var.	Fixed Term	Coeff.	χ^2^	DF	*p*‐value
Pronotum Width (*N* = 253)	M + F	Week	0.020	Intercept	−27.18			
		Week/trap	0.177	Sex (males): Position	[0.02]	2.92	1	.088
		Residual	0.331	Sex (males)	−0.23	6.30	1	.**012**
				Population Density	[−0.33]	0.49	1	.484
				Position	0.02	5.06	1	.**024**
Pronotum Width (*N* = 161)	F	Week	0.003	Intercept	8.04			
		Week/trap	0.203	Position	[0.01]	0.40	1	.529
		Residual	0.352	Population Density	[<−0.11]	0.05	1	.829
Pronotum Width (*N* = 92)	M	Week	0.048	Intercept	−66.62			
		Week/trap	0.134	Position	0.04	9.88	1	.**002**
		Residual	0.264	Population Density	[−0.65]	0.91	1	.341

Note

Sex, position along the expansion front (position), and population density (the number of beetles caught per trap and trapping day for each trap row) were used as fixed terms. Coefficients (Coeff.) in square brackets belong to nonsignificant terms just before dropping those terms from the model. Bold *p*‐values denote significant terms. Variance (Var.) of the random terms “Week” and “Week/trap” (the trap from which individuals were collected nested within the week of collection) and residuals are presented.

**TABLE 2:** Summary of test statistics from LMMs with body condition as a response in males and females (M + F), females alone (F), and males alone (M)

Response variable	Sex	Random term	Var.	Fixed term	Coeff.	χ^2^	DF	*p*‐value
Body Condition (*N* = 253)	M + F	Week	0.002	Intercept	0.02			
		Week/trap	0.002	Sex (males): Position	[<−0.01]	0.01	1	.921
		Residual	0.005	Population Density	−0.14	10.02	1	.**002**
				Sex (males)	[<0.01]	0.01	1	.923
				Position	[<−0.01]	0.72	1	.398
Body Condition (*N* = 161)	F	Week	0.002	Intercept	−0.01			
		Week/trap	0.002	Population Density	[<−0.12]	3.55	1	.060
		Residual	0.007	Position	[<−0.01]	0.25	1	.619
Body Condition (*N* = 92)	M	Week	<0.001	Intercept	0.03			
		Week/trap	0.002	Population Density	−0.17	9.93	1	.**002**
		Residual	0.002	Position	[<−0.01]	1.02	1	.313

Note

Sex, position along the expansion front (position), and population density (the number of beetles caught per trap and trapping day for each trap row) are used as fixed terms. Coefficients (Coeff.) in square brackets belong to nonsignificant terms just before dropping those terms from the model. Bold *p*‐values denote significant terms. Variance (Var.) of the random terms “Week” and “Week/trap” (the trap from which individuals were collected nested within the week of collection) and residuals are presented. Bold *p*‐values denote significant terms.

The sex specificity of the effect of position along the expansion front on individual body size and condition was determined by using two additional LMMs per response variable using only female or male data. The structure of the models was the same as above excluding “sex” and its interaction with the position along the expansion front as explanatory variables.

Because a significant negative relationship was found between population density and position along the expansion front, population density might mask the effects of position along the expansion front. Thus, body size and body condition LMMs, for male and female combined data, female data alone, and male data alone, were rerun without population density as an explanatory variable. Removal of population density from the maximal models for body size (Table 1) or body condition (Table 2) did not qualitatively change our results (Tables S2 and S3).

## 3 RESULTS

**FIGURE 1:**
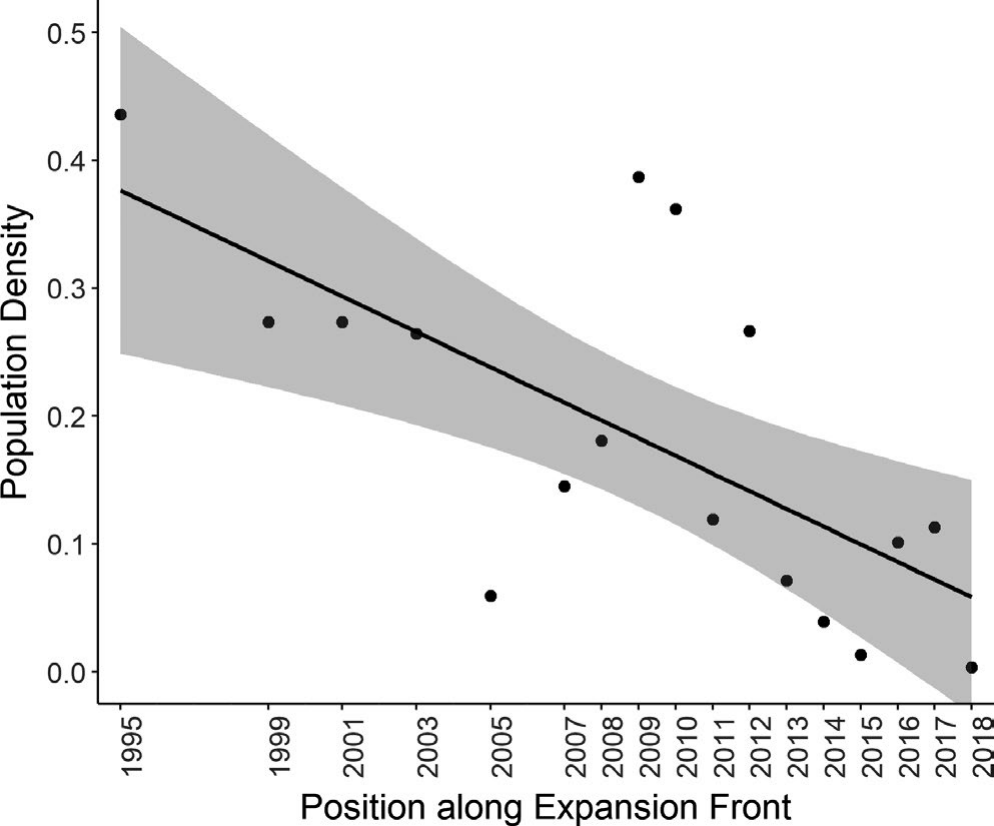
The relationship between *C. hortensis* population density and position along the expansion front (*N* = 17). Years denote the previous locations of the westerly range edge of *C. hortensis* in that year, such that 2018 is the range edge in 2018. Population density is the mean number of beetles per trap and trapping day across a trap row. Predicted line is fitted using outputs from LM estimates. 95% confidence interval is shown in gray

As predicted, population density decreased with proximity to the *C. hortensis* expansion front in the Lüneburger Heide (LM; *R* ^2^ = 0.406, *F*_1,15_ = 10.250, *p* =.006; Figure 1). The female‐to‐male sex ratio was negatively correlated with position along the expansion front (*R*_S_ = −0.492, *p* =.044, *N* = 17), meaning that proportionally fewer females were found at the range edge.

Female *C. hortensis* were larger than males (Table 1). Female pronotum width was 8.0 ± 0.1 mm (mean ±SE) (range: 5.8 – 9.8 mm), while male pronotum width was 7.8 ± 0.1 mm (range: 5.8 – 9.2 mm). Males and females did not significantly differ in their body condition (Table 2).

**FIGURE 2:**
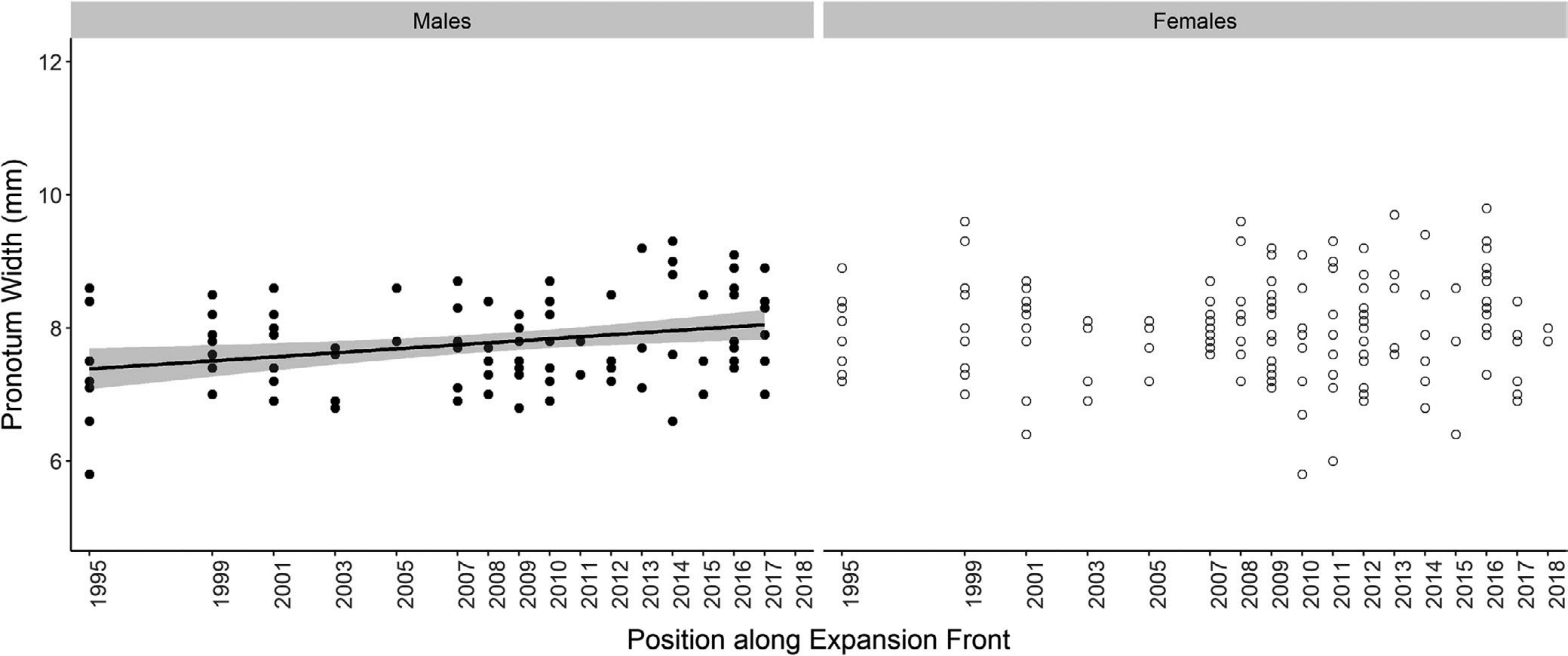
The relationship between individual *C. hortensis* body size and position along the expansion front in males (*N* = 92) and females (*N* = 161). Years denote the previous locations of the westerly range edge of *C. hortensis* in that year, such that 2018 is the range edge at the time of study in 2018. Predicted line is fitted using outputs from LMM estimates. 95% confidence interval is shown in gray

The body size of all beetles (Table 1) and male beetles (Table 1; Figure 2) increased toward the range edge. However, female body size did not significantly change with position along the expansion front (Table 1; Figure 2). There was a marginally significant trend for an interactive effect of sex upon the relationship between body size and position along the expansion front. Body size was independent of population density in both sexes (Table 1). Male front tibia length, male mid‐tibia length, and female hind tibia length were positively correlated with body size (Table S1).

**FIGURE 3:**
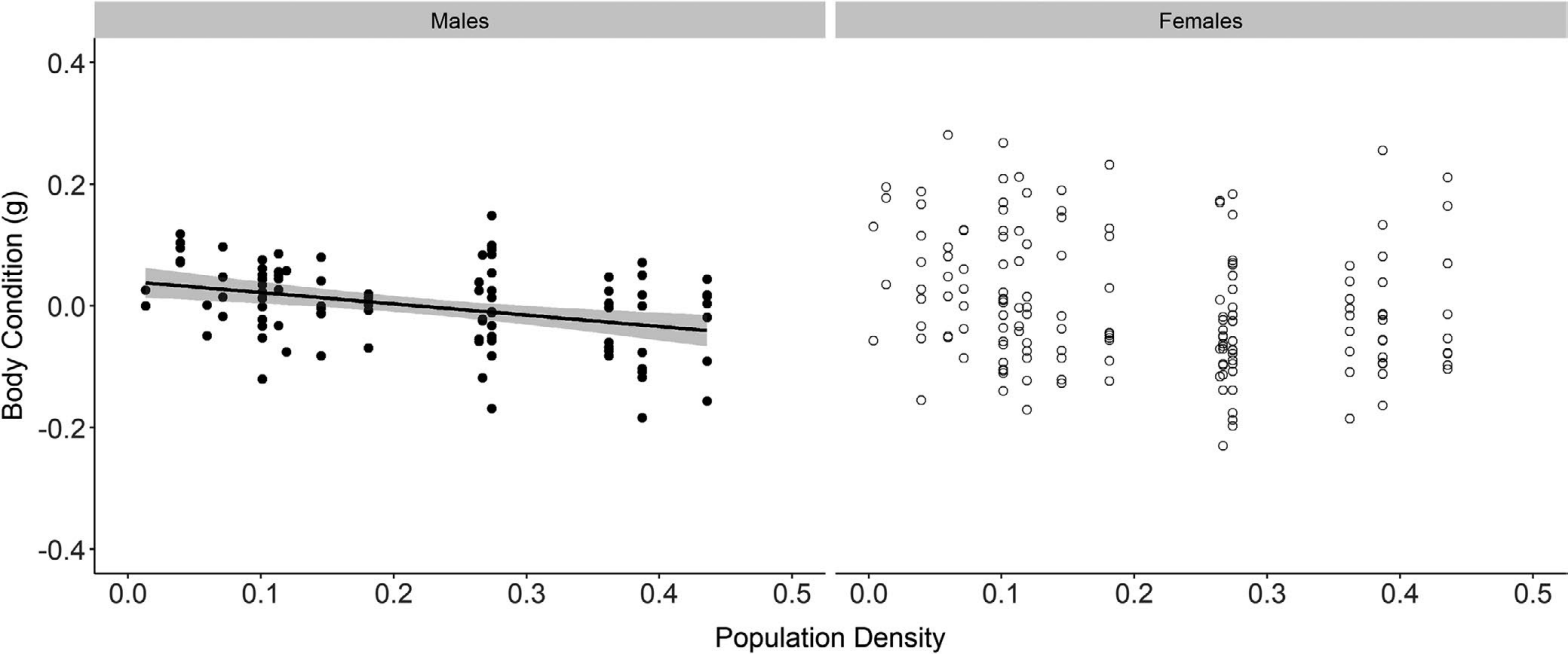
The relationship between individual *C. hortensis* body condition and population density in males (*N* = 92) and females (*N* = 161). Population density is the mean number of beetles per trap and trapping day across a trap row. Predicted line is fitted using outputs from LMM estimates. 95% confidence interval is shown in gray

There was no significant relationship between body condition and position along the expansion front for either males or females (Table 2). However, the body condition of all beetles (Table 2) and male beetles alone (Table 2; Figure 3) increased with decreasing population density. Female body condition, however, was independent of population density (Table 2; Figure 3).

## 4 DISCUSSION

We investigated variation of traits associated with morphology across an expansion front on a sex‐specific basis in the ground beetle *Carabus hortensis*. As we hypothesized, body size increased with proximity to the range edge in males but not in females. Although body condition did not increase with proximity to the range edge in either sex as we had predicted, male body condition alone improved with decreasing population density, which was lowest at the edge of the *C. hortensis* range. This may indicate that male body condition was generally higher in areas with low intraspecific competition. Sex differences in the relationships between body size and position along the expansion front, and between body condition and population density, may be rooted in sex differences in activity. Together, our findings provide evidence of sex‐specific relationships between morphology and position along an expansion front.

Consistent with our predictions, male but not female *C. hortensis* were larger at the range edge than toward the core of the species’ range. Such sex‐specific changes in body size distribution across the *C. hortensis* range could occur if divergent selection pressures act upon males and females to produce differences in traits associated with morphology between the sexes (Bateman, 1948; Darwin, 1871; Trivers, 1972) that alter movement capacity. Moreover, differences in male and female activity could underpin the differences between the sexes in the distribution of body sizes across the *C. hortensis* range. Male *C. hortensis* are the more active sex (Szyszko et al., 2004); if higher activity levels in males support a male‐led range expansion, male body size may change more strongly than female body size across the *C. hortensis* range. That the female‐to‐male sex ratio declined with proximity to the range edge supports that range expansion by *C. hortensis* in the Lüneburger Heide may be mainly male‐led. Increases of male body size toward the *C. hortensis* range edge may be further reinforced through sexual selection (*e.g*., Hengeveld & Haeck, 1982); where population densities and mate availability are lower at the range edge, males may be sexually selected for larger body size, which may improve movement capacity and related mate searching ability (*e.g*., Arnold et al., 2017; Zollikofer, 1994). Again, this hypothesis is supported by our findings that the female‐to‐male sex ratio decreased toward the range edge. To understand the mechanisms underlying sex‐specific trait differentiation across species’ shifting ranges, more studies investigating species with different mating systems are needed.

In line with previous studies of intraspecific competition effects on carabid beetles (Lenski, 1984), male *C. hortensis* from trap rows with lower population densities had a better body condition. Female body condition, however, was unrelated to population density. Population density influences body condition both by altering competition for resources (Iba et al., 1995) and by influencing individual activity level (Le Galliard et al., 2015; Tuda & Shima, 2002) and associated energy expenditure. Consequently, sex differences in the relationships between population density and body condition may arise if there are (a) sex differences in activity level and/or (b) sex differences in the motivations for activity, because population density will influence activity (and associated energy expenditure) differentially between the sexes. For instance, because male *C. hortensis* activity is likely associated with reproductive success, males may be similarly motivated to be active irrespective of whether they are in areas of high or low population density. This may create an imbalance between energy consumption and expenditure under high population densities, because males living under high population densities will experience high intraspecific competition, leading to lower resource availability and associated energy intake than males living under low population densities that have similar energy expenditure levels. In contrast, female *C. hortensis* may adjust their activity levels to match the population density and related resource availability. Our results suggest that sex can be an important factor in determining how population density will relate to body condition, where males and females differ in activity level. Further investigations into the effects of population density on body condition in systems where males and females differ substantially in their behaviors and life histories could help to reinforce our findings.

Very few studies have investigated patterns in sex‐specific traits associated with morphology across invertebrates’ shifting ranges and, thus far, results are mixed. Some studies report that differential morphological traits between the sexes increased with proximity to the range edge (Hughes et al., 2003), while others state that the same morphological traits increased toward the range edge in both sexes, but with a stronger effect in one sex over the other (Laparie et al., 2013). We believe that our study is the first to report the increase in size of morphological traits associated with movement toward a species’ range edge in just one sex, in an insect. Some vertebrate studies of morphological changes across species’ range expansions are in line with our own, reporting that only male morphological traits increase with proximity to the range edge of an expanding or shifting range (Bodden & Puschendorf, 2019; Campbell & Echternacht, 2003; Gunnarsson et al., 2012). Conversely, for other vertebrates, traits that increased with proximity to the range edge did so in both sexes, but the effect was stronger in males than females (Padilla et al., 2019; Simberloff et al., 2000). In general, it appears that traits such as body size, wing length, and muscle mass (*i.e*., traits that improve movement propensity) are most likely to increase with proximity to the range edge in males. Still, there are too few studies to draw conclusions upon the role of sex in the distribution of morphological traits across species’ ranges, especially in insects. Further work evaluating sex‐specific patterns in trait distribution across species’ shifting ranges in a range of species with different mating systems will help to further this field.

## 5 CONCLUSIONS

Ours is the first study of an insect species to report that morphological traits associated with movement may change across a species’ range in just one sex. We demonstrated that body size increased across the expansion front in male but not female *C. hortensis* beetles. Males at the range edge of the expansion front were larger than conspecifics farther back in the species’ range. Furthermore, male body condition declined with increasing population density. In contrast, we found no significant relationship between female body size and position along the expansion front and no significant relationship between female body condition and population density. We argue that the observed differences between male and female *C. hortensis* may be linked to differences in the reproductive biology of the sexes and sex differences in activity level, leading to differential distributions of male and female body size in space (Bateman, 1948; Trivers, 1972). Our results move the field forwards, demonstrating that sex and sex differences in behavior play an important role in determining the distribution of morphological traits across species’ shifting ranges.

## CONFLICT OF INTEREST

The authors declare that there is no conflict of interest.

## ETHICS

The study was carried out under permits from the Heidekreis and Harburg nature conservation authorities and the Lower Saxon State Department for Waterway, Coastal and Nature Conservation authorities (number: H72.2220212019) which allowed entry into the Lüneburger Heide nature reserve and collection of beetles of the genus *Carabus* therein, respectively.

## AUTHOR CONTRIBUTION

**Elisabeth Yarwood:** Data curation (lead); Formal analysis (lead); Investigation (equal); Methodology (supporting); Visualization (lead); Writing‐original draft (lead). **Claudia Drees:** Conceptualization (equal); Methodology (equal); Project administration (equal); Resources (equal); Supervision (equal); Writing‐review & editing (equal). **Jeremy E Niven:** Formal analysis (supporting); Supervision (equal); Writing‐review & editing (equal). **Marisa Gawel:** Data curation (supporting); Investigation (equal); Methodology (supporting). **Wiebke Schuett** **:** Conceptualization (equal); Formal analysis (supporting); Funding acquisition (lead); Methodology (equal); Project administration (equal); Resources (equal); Supervision (lead); Writing‐review & editing (lead).

## Supporting information

Supplementary Material

## Data Availability

Data presented are provided in Dryad dataset link: https://doi.org/10.5061/dryad.v41ns1rvx
