# Supplementary material for: Sex differences in morphology across an expanding range edge in the flightless ground beetle, Carabus hortensis
Source: Ecol Evol. 2021 Jul 1;11(15):9949–57. doi: 10.1002/ece3.7593 (PMC8328432; doi:10.1002/ece3.7593)
Supplement: Supplementary file 1 — Supplementary Material [file ECE3-11-9949-s001.docx]

# Appendix

**Table S1. Sex-specific Spearman’s rank correlations between morphological traits measured in Carabus hortensis.** Correlations using data from females alone (F) and data from males alone (M) are presented. Bold p-values denote significant correlations. Bold R_s_ values denote strong correlations (R > 0.7). Asterisks on p-values denote which correlations remain significant following False Discovery Rate testing (Benjamini & Hochberg, 1995).

| ***Sex*** | ***1^st^ Variable*** | ***2^nd^ Variable*** | ***R_s_*** | ***p-value*** | ***N*** |
| --- | --- | --- | --- | --- | --- |
| F | Body Condition (g) | Body Mass (g) | **0.936** | **<0.001*** | 161 |
| F | Pronotum Width (mm) | Body Mass (g) | 0.275 | **<0.001*** | 161 |
| F | Body Condition (g) | Pronotum Width (mm) | -0.035 | 0.661 | 161 |
| F | Pronotum Width (mm) | Front Femur | 0.239 | 0.118 | 44 |
| F | Pronotum Width (mm) | Mid Femur | 0.199 | 0.195 | 44 |
| F | Pronotum Width (mm) | Hind Femur | 0.178 | 0.207 | 44 |
| F | Pronotum Width (mm) | Front Tibia | 0.157 | 0.310 | 44 |
| F | Pronotum Width (mm) | Mid Tibia | 0.165 | 0.285 | 44 |
| F | Pronotum Width (mm) | Hind Tibia | 0.385 | **0.010*** | 44 |
| M | Body Condition (g) | Body Mass (g) | **0.941** | **<0.001*** | 92 |
| M | Pronotum Width (mm) | Body Mass (g) | 0.267 | **0.010*** | 92 |
| M | Body Condition (g) | Pronotum Width (mm) | -0.018 | 0.883 | 92 |
| M | Pronotum Width (mm) | Front Femur | -0.178 | 0.427 | 22 |
| M | Pronotum Width (mm) | Mid Femur | 0.182 | 0.418 | 22 |
| M | Pronotum Width (mm) | Hind Femur | 0.220 | 0.324 | 22 |
| M | Pronotum Width (mm) | Front Tibia | 0.610 | **0.003*** | 22 |
| M | Pronotum Width (mm) | Mid Tibia | 0.547 | **0.008*** | 22 |
| M | Pronotum Width (mm) | Hind Tibia | 0.268 | 0.228 | 22 |

**Table S2. Summary of test statistics from LMMs with the pronotum width as a proxy for body size as a response** **in males and females (M + F), females alone (F) and males alone (M).** Sex and position along the expansion front (Position) were used as fixed terms. Coefficients (Coeff.) in square brackets belong to non-significant terms just before dropping those terms from the model. Bold p-values denote significant terms. Variance (Var.) of the random terms ‘Week’ and ‘Week/trap’ (the trap from which individuals were collected nested within the week of collection) and residuals are presented.

| ***Sex*** | ***Random***  ***Term*** | ***Var.*** | ***Fixed Term*** | ***Coeff.*** | **χ*^2^*** | ***DF*** | ***p-value*** |
| --- | --- | --- | --- | --- | --- | --- | --- |
| M + F | Week | 0.020 | Intercept | -27.18 |  |  |  |
| (*N* = 253) | Week/trap | 0.177 | Sex (males): Position | [0.02] | 2.94 | 1 | 0.086 |
|  | Residual | 0.308 | Sex (males) | -0.23 | 6.30 | 1 | **0.012** |
|  |  |  | Position | 0.02 | 5.06 | 1 | **0.024** |
| F | Week | 0.003 | Intercept | 8.04 |  |  |  |
| (*N* = 161) | Week/trap | 0.203 | Position | [0.01] | 0.40 | 1 | 0.529 |
|  | Residual | 0.352 |  |  |  |  |  |
| M | Week | 0.048 | Intercept | -66.62 |  |  |  |
| (*N* = 92) | Week/trap | 0.134 | Position | 0.04 | 9.88 | 1 | **0.002** |
|  | Residual | 0.264 |  |  |  |  |  |

| ***Sex*** | ***Random***  ***Term*** | ***Var.*** | ***Fixed Term*** | ***Coeff.*** | **χ*^2^*** | ***DF*** | ***p-value*** |
| --- | --- | --- | --- | --- | --- | --- | --- |
| M+F | Week | 0.002 | Intercept | -0.02 |  |  |  |
| (*N* = 253) | Week/trap | 0.002 | Sex (males): Position | [<-0.01] | 0.01 | 1 | 0.928 |
|  | Residual | 0.005 | Sex (males) | [<-0.01] | 0.01 | 1 | 0.936 |
|  |  |  | Position | [<0.01] | 1.77 | 1 | 0.183 |
| F | Week | 0.002 | Intercept | -0.01 |  |  |  |
| (*N* = 161) | Week/trap | 0.002 | Position | [<0.01] | 0.59 | 1 | 0.443 |
|  | Residual | 0.007 |  |  |  |  |  |
| M | Week | <0.001 | Intercept | -0.02 |  |  |  |
| (*N* = 92) | Week/trap | 0.002 | Position | [<0.01] | 1.53 | 1 | 0.216 |
|  | Residual | 0.002 |  |  |  |  |  |

**Table S3. Summary of test statistics from LMMs with body condition as a response** **in males and females (M + F), females alone (F) and males alone (M).** Sex and position along the expansion front (Position) are used as fixed terms. Coefficients (Coeff.) in square brackets belong to non-significant terms just before dropping those terms from the model. Bold p-values denote significant terms. Variance (Var.) of the random terms ‘Week’ and ‘Week/trap’ (the trap from which individuals were collected nested within the week of collection) and residuals are presented. Bold p-values denote significant terms.

# References

Benjamini, Y., & Hochberg, Y. (1995). Controlling the false discovery rate: a practical and powerful approach to multiple testing. *Journal of the Royal Statistical Society: Series B (Methodological)*, *57*(1), 289–300.
